# A Novel Selective Sphingosine Kinase 2 Inhibitor, HWG-35D, Ameliorates the Severity of Imiquimod-Induced Psoriasis Model by Blocking Th17 Differentiation of Naïve CD4 T Lymphocytes

**DOI:** 10.3390/ijms21218371

**Published:** 2020-11-08

**Authors:** Sun-Hye Shin, Hee-Yeon Kim, Hee-Soo Yoon, Woo-Jae Park, David R. Adams, Nigel J. Pyne, Susan Pyne, Joo-Won Park

**Affiliations:** 1 Department of Biochemistry, College of Medicine, Ewha Womans University, Seoul 07804, Korea; s1sunhye@naver.com (S.-H.S.); heeyeon1432@gmail.com (H.-Y.K.); gltn129@naver.com (H.-S.Y.); 2 Department of Biochemistry, College of Medicine, Gachon University, Incheon 21999, Korea; ooze@gachon.ac.kr; 3 School of Engineering & Physical Sciences, Heriot-Watt University, Edinburgh EH14 4AS, UK; D.R.Adams@hw.ac.uk; 4 Strathclyde Institute of Pharmacy and Biomedical Sciences, University of Strathclyde, Glasgow G4 0RE, UK; n.j.pyne@strath.ac.uk (N.J.P.); susan.pyne@strath.ac.uk (S.P.)

**Keywords:** psoriasis, HWG-35D, sphingosine kinase, sphingosine-1-phosphate, T helper type 17 differentiation

## Abstract

Sphingosine kinases (SK) catalyze the phosphorylation of sphingosine to generate sphingosine-1-phosphate. Two isoforms of SK (SK1 and SK2) exist in mammals. Previously, we showed the beneficial effects of SK2 inhibition, using ABC294640, in a psoriasis mouse model. However, ABC294640 also induces the degradation of SK1 and dihydroceramide desaturase 1 (DES1). Considering these additional effects of ABC294640, we re-examined the efficacy of SK2 inhibition in an IMQ-induced psoriasis mouse model using a novel SK2 inhibitor, HWG-35D, which exhibits nM potency and 100-fold selectivity for SK2 over SK1. Topical application of HWG-35D ameliorated IMQ-induced skin lesions and normalized the serum interleukin-17A levels elevated by IMQ. Application of HWG-35D also decreased skin mRNA levels of interleukin-17A, K6 and K16 genes induced by IMQ. Consistent with the previous data using ABC294640, HWG-35D also blocked T helper type 17 differentiation of naïve CD4^+^ T cells with concomitant reduction of SOCS1. Importantly, HWG-35D did not affect SK1 or DES1 expression levels. These results reaffirm an important role of SK2 in the T helper type 17 response and suggest that highly selective and potent SK2 inhibitors such as HWG-35D might be of therapeutic use for the treatment of psoriasis.

## 1. Introduction

Psoriasis is a chronic inflammatory dermatologic disease, affecting 2–3% of the adult population and 0.05–2.20% of children [1,2]. Patients with psoriasis vulgaris present with scaly, erythematous plaques on the skin, and the skin lesions are usually accompanied by an infiltration of immune cells and accelerated proliferation of keratinocytes and endothelial cells [3]. Recent advances have enhanced our understanding of disease mechanisms in psoriasis pathogenesis; psoriasis is generally considered as an autoimmune disease and roles of T cells are especially important for disease pathogenesis [2]. T-helper (Th) cells can be classified into Th1, Th2, and Th17 cells; Th cells can also be regulated by regulatory T cells [4]. Briefly, Th1 cells, which produce interleukin (IL)-2 and interferon (IFN)-γ, are mainly involved in cellular immunity whereas Th2 cells, which produce IL-4, IL-5 and IL-13, are involved in humoral immunity [4]. Th17 cells, which produce IL-17A, IL-17F, IL-21 and IL-22, play important roles for the induction of inflammation and have been implicated in pathogenic mechanisms in various autoimmune diseases [4,5]. Initially, psoriasis was considered to be caused by a Th1 response with IFN-γ and IL-12 as signature cytokines. However, recently the key role of both the innate immune cells in the affected tissue and adaptive Th17 cell responses have been identified as the major mechanisms [6]. Polymorphisms of genes encoding Th17-associated factors, such as IL-23, IL-23 receptor and relevant signaling molecules including suppressor of cytokine signaling (SOCS) 1 and signal transducers and activators of transcription 3 (STAT3) are associated with psoriasis [3,7,8]. In addition, the antimicrobial peptide LL37 has been reported as a putative autoantigen in psoriasis with the potential to promote secretion of Th17 cytokines by reactive CD4+ T cells [3,9]. 

Sphingolipids are a diverse class of lipids comprising a backbone of sphingoid bases derived from aliphatic amino alcohols such as sphinganine and sphingosine [10]. The most abundant mammalian sphingoid base is the 18 carbon dihydroxy amino alkene, sphingosine and its saturated precursor is sphinganine [10]. Sphinganine or sphingosine can be phosphorylated at the 1-OH group by sphingosine kinase (SK) enzymes to produce sphinganine-1-phosphate or sphingosine-1-phosphate (S1P) [11]. Two isoforms of SK, SK1 and SK2, exist in mammals [12]. These enzymes reside in distinct subcellular compartments and regulate different subcellular pools of S1P [12]. In the absence of stimulation, SK1 is a predominantly cytosolic enzyme, but it moves to the plasma membrane upon stimulation [13]. In contrast, SK2 has a nuclear localization signal in the N-terminus and a nuclear export signal within the C-terminal domain and may shuttle between the cytoplasm and the nucleus, but is also reported in other subcellular locations [13]. In the nucleus, SK2 can interact with histone H3–histone deacetylase 1/2 complexes; S1P produced by SK2 inhibits histone H3–histone deacetylase 1/2 complex-mediated deacetylation of histone H3 and promotes the transcription of several genes, such as cyclin-dependent kinase inhibitor p21 and the transcriptional regulator c-fos [14]. Despite some redundancy in function, these two isoforms display distinct biochemical properties and inhibitor sensitivities [12]. A number of SK2 inhibitors have been generated but, until recently, available inhibitors have exhibited only modest potency and/or selectivity over SK1 and, in some cases, have additional effects on other protein targets [12]. For example, the much-studied SK2 inhibitor, ABC294640, also binds to the estrogen receptor and functions as a partial antagonist similar to tamoxifen [15]. In addition, ABC294640 induces the ubiquitin-proteasomal degradation of SK1 and dihydroceramide desaturase 1 (DES1) [16]. Some of us recently performed a ligand-based structure activity relationship study to highlight the key determinants for switching between SK1- and SK2-selective inhibition, leading to the design of the novel SK2 inhibitor, HWG-35D (compound 55 in Adams et al. [17]), which exhibits an IC_50_ of 41 nM and 100-fold selectivity for SK2 over SK1 [17].

S1P is a bioactive signaling lipid that modulates cell growth, apoptosis and immune reactions and is now regarded as a crucial regulator of many physiological and pathophysiological processes, including cancer, atherosclerosis, diabetes and osteoporosis [18,19]. Recently, several human studies have reported a correlation of psoriasis with S1P levels; circulating S1P levels were significantly increased in psoriatic patients compared to the healthy control group [19], especially in severe cases [20]. Furthermore, S1P modulation using various chemicals was effective in ameliorating psoriasis symptoms in animal psoriasis models [21,22,23]. For instance, IMMH002, an orally active S1P_1_ receptor modulator, desensitized egress of peripheral pathogenic lymphocytes from secondary lymphoid organs and the thymus and significantly relieved psoriasis skin damage in both imiquimod (IMQ, a toll-like receptor 7/8 agonist)-treated mouse models and in guinea pigs with propranolol-induced psoriasis-like skin lesions [23]. Recently, our group showed the effect of SK2 inhibition in a psoriasis mouse model using the modestly potent SK2 inhibitor, ABC294640, which diminished Th17 differentiation of naïve CD4^+^ T cells with concomitant reduction in SOCS1 mRNA levels [21]. Due to the potential complicating effect of ABC294640 on SK1 and DES1 [15,24], we have now re-examined the efficacy of SK2 inhibition in IMQ-induced psoriasis mouse models using a novel specific SK2 inhibitor, HWG-35D [17].

## 2. Results

### 2.1. Neither SK1 nor DES1 Protein Levels Were Altered by a Novel Selective SK2 Inhibitor, HWG-35D

**Figure 1:**
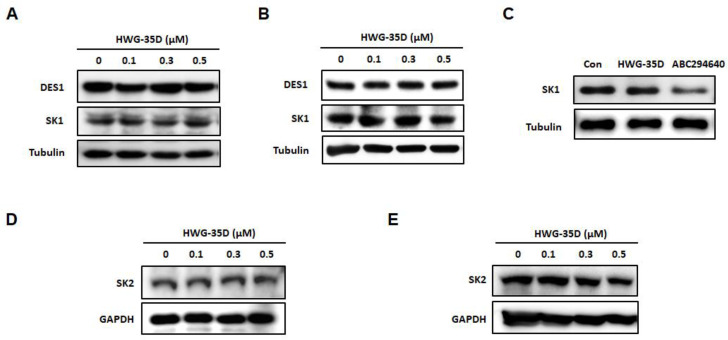
HWG-35D does not alter dihydroceramide desaturase 1 (DES1), sphingosine kinase (SK) 1, and SK2 protein levels. (**A** and **D**) MCF-7 and (**B** and **E**) NIH3T3 cells were incubated with HWG-35D at the indicated concentrations for 24 h. (**C**) Total CD4^+^ T cells isolated from murine spleen were treated with HWG-35D (300 nM) or with ABC294640 (25 μM) for 24 h. Western blot analysis was performed to examine the degradation of DES1, SK1 and SK2. The representative images are shown of three independent experiments.

The SK2 inhibitor, ABC294640 has been reported to induce the ubiquitin-proteasomal degradation of SK1 and DES1 [16,17]. In contrast, another SK2 inhibitor, (R)-FTY720 methyl ether (ROMe), does not induce the proteasomal degradation of SK1 [25]. We therefore, examined the effects of a novel potent and selective SK2 inhibitor, HWG-35D, on SK1 and DES1 expression levels. HWG-35D (0­–500 nM) did not alter either SK1 or DES1 protein expression in either human or murine cells (Figure 1A,B). In addition, HWG-35D (300 nM) did not decrease SK1 protein levels in total CD4^+^ T cells isolated from mouse spleen, whereas ABC294640 (25 μM) reduced SK1 protein levels as reported previously in other cell types [24] (Figure 1C). DES1 was not detected in total CD4^+^ T cells isolated from mouse spleen. Taken together, these results are consistent with HWG-35D, acting as a selective SK2 inhibitor with no effect on SK1 or DES1 levels. In addition, HWG-35D did not induce SK2 degradation in either human or murine cells (Figure 1D,E), suggesting HWG-35D as a selective SK2 inhibitor, which does not affect its protein levels.

### 2.2. Selective SK2 Inhibition Using HWG-35D Improves Severity of Psoriasis-Like Skin Lesions in a Murine Model of Psoriasis With the Dosage of 25 µG/40 µL Surfactant

**Figure 2:**
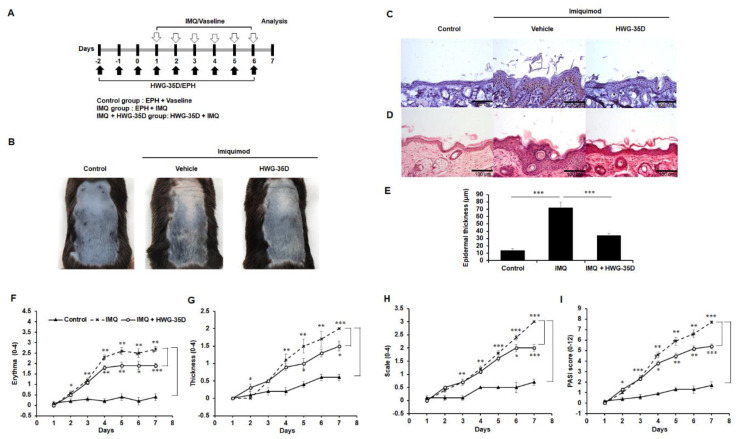
Topical application of HWG-35D reduces psoriasis-like skin symptoms induced by imiquimod. (**A**) Study plan: C57BL/6 mice were treated daily with imiquimod (IMQ) cream or the control vehicle cream on the shaved back for 6 consecutive days. HWG-35D, a specific sphingosine kinase (SK) 2 inhibitor, was treated from 3 days before IMQ application with a dosage of 25 µg/40 µL surfactant (100% ethanol, propylene glycol, and H_2_O (EPH) at 2:1:1 (*v/v/v*)). (**B**) Phenotypical presentation of mouse back skin after 6 days of treatment. (**C**) Immunohistochemical staining of paraffin-embedded skin tissue sections for Ki-67 expression. (**D**) Haematoxylin and eosin-stained skin tissue sections, and (**E**) quantification of epidermis in the sections. The severity of (**F**) erythema, (**G**) thickening and (**H**) scaling were scored using the clinical Psoriasis Area and Severity Index (PASI), as indicated in the previous study [21]. (**I**) The cumulative PASI score indicates the comprehensive severity of skin inflammation. Data are presented as mean ± S.E.M. values (*n* = 5). * *p* < 0.05, ** *p* < 0.01, *** *p* < 0.001. The representative images are shown of three independent experiments (magnification ×20).

Previously, topical application of ABC294640 mitigated symptoms of psoriasis-like skin lesions in a mouse model of psoriasis [21]. Considering the complicating off-target effects of ABC294640 [15,24], we re-investigated the effects of topical SK2 inhibition in psoriasis-like skin disease using the newly developed selective SK2 inhibitor, HWG-35D [17] and using the same dosage used for ABC294640 to enable a direct comparison [17,21]. To determine the absolute bioavailability of HWG-35D in vivo, a pharmacokinetic study of HWG-35D must be performed. In the absence of pharmacokinetic data, we used the same dosage used for ABC294640. HWG-35D was topically applied as a pre-treatment for 3 days to decrease S1P levels generated via SK2; then IMQ was administered with HWG-35D to establish the impact of HWG-35D on formation of the psoriasis-like skin lesions for 6 days (Figure 2A). As reported previously [21,26], topical application of IMQ in mouse back skin resulted in a psoriasis-like skin inflammation with accompanying symptoms such as erythema and scale formation (Figure 2B). Consistent with ABC294640 [21], SK2 inhibition using HWG-35D also improved the psoriasis-like skin phenotype of mice (Figure 2B). The Ki-67 staining showed a reduced proliferative response in the HWG-35D-treated IMQ epidermis compared with the Vaseline-treated IMQ group (Figure 2C). The H&E staining also showed the reduction of epidermal thickness in the HWG-35D-treated group (Figure 2D and E). Daily skin changes were monitored using the PASI scoring system, which grades the psoriasis plaques for their combined redness (Figure 2F), thickness (Figure 2G) and scaling (Figure 2H), as previously described [21]. The PASI score of IMQ-treated group started to increase on the second day of IMQ application, and the mean ± S.E.M. PASI score was increased to 7.7 ± 0.27 on the seventh day of IMQ application (Figure 2I). In the HWG-35D-treatment group, the mean ± S.E.M. PASI score was 5.4 ± 0.74 on the seventh day of IMQ application, and the differences between the HWG-35D-treated IMQ group and the Vaseline-treated IMQ group were statistically significant from the fourth day of IMQ application (Figure 2I). These results confirm the protective effect of SK2 inhibition with HWG-35D in psoriasis-like skin disease.

### 2.3. Systemic Immune Reaction Caused by IMQ Was Diminished With Topical Application of HWG-35D

**Figure 3:**
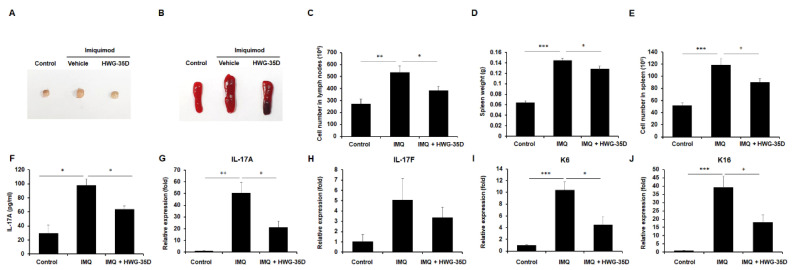
HWG-35D treatment normalizes both systemic and local immune response induced by IMQ treatment. Gross morphologic features of (**A**) inguinal lymph node and (**B**) spleen. (**C**) Total cell numbers in inguinal lymph nodes were analyzed. (**D**) Spleen weights and (**E**) total cell numbers of the spleen were examined. (**F**) Serum IL-17A levels were investigated using ELISA. The mRNA levels of (**G**) IL-17A, (**H**) IL-17F, (**I**) keratin 6 (K6), and (**J**) keratin 16 (K16) were analyzed using real-time PCR. Data are mean ± S.E.M. values (*n* = 5). * *p* < 0.05, ** *p* < 0.01, *** *p* < 0.001.

The systemic immune response plays an integral role in psoriasis and cytokines released from T cells and dendritic cells mediate the effects on keratinocytes to amplify psoriatic inflammation [27]. Therefore, the size and cellularity of lymphoid organs was evaluated to investigate whether topical application of HWG-35D affects the systemic immune response induced by IMQ (Figure 3). The size of the spleen and the inguinal lymph nodes were increased in the IMQ-treated group compared with the Vaseline-treated group after 7 days of application (Figure 3A,B). Topical application of HWG-35D decreased the IMQ-induced inguinal lymph node and spleen enlargement compared with the Vaseline-treated group (Figure 3A,B). Consistent with the size of lymphoid organs, the mean cell numbers in the inguinal lymph nodes were significantly increased upon IMQ application, and HWG-35D administration decreased the mean cell numbers in the inguinal lymph nodes (Figure 3C). Similarly, the average spleen weights and splenocyte numbers were increased upon IMQ application but were significantly diminished in the HWG-35D-treated group compared with the Vaseline-treated group (Figure 3D,E). Since psoriasis is considered to be an IL-17A-driven disease [28], serum IL-17A levels were measured. As expected, IMQ treatment increased serum IL-17A levels, and topical application of HWG-35D significantly reduced serum IL-17A levels (Figure 3F). These data indicate that topical application of HWG-35D diminishes IMQ-induced systemic immune reaction, including Th17 induction.

### 2.4. Topical HWG-35D Application Normalises mRNA Levels of Genes Associated With Th17 Response and Keratinization

The Th17-mediated immune response plays a central role in the pathogenesis of psoriasis [28], and abnormal keratinization is one of the representative features of psoriasis. Thus, alteration of gene expression involved in the Th17 response and keratinization was evaluated in the skin lesion. In accordance with the serum results (Figure 3F), IL-17A mRNA levels were elevated in IMQ-treated skin samples, and topical application of HWG-35D markedly suppressed IL-17 mRNA levels (Figure 3G). Despite the similar tendency of IL-17F mRNA levels with IL-17A mRNA levels, the alteration was not statistically significant (Figure 3H). Keratins K6 and K16 have been recognized as barrier alarms that are rapidly induced in stressed keratinocytes at the suprabasal layers of the epidermis within hours after injury [29] and are used as markers of keratinocyte hyper-proliferation [30]. IMQ treatment markedly increased expression of K6 and K16 keratin genes and topical application of HWG-35D markedly normalised the mRNA levels for these genes (Figure 3I and J). These data indicate that SK2 inhibition with HWG-35D ameliorates the regional Th17-mediated immune response and abnormal keratinization induced by IMQ treatment.

### 2.5. Inhibition of SK2 With HWG-35D Blocks Th17 Polarization In Vitro

**Figure 4:**
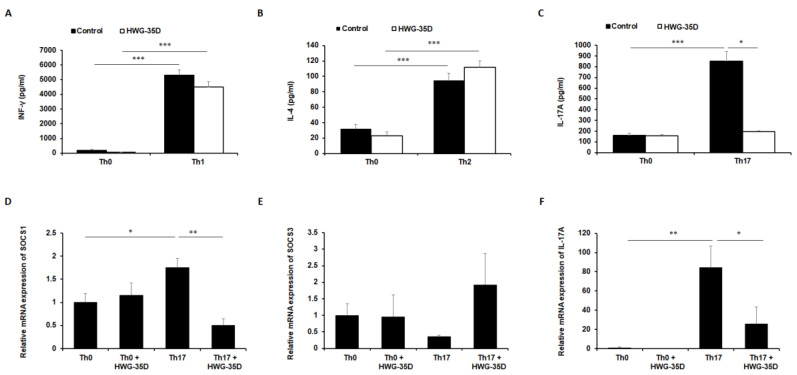
Th17 differentiation of naïve CD4+ T cells is inhibited by HWG-35D. Naïve CD4^+^ T cells isolated from the mouse spleen were polarized using differentiating media into Th1, Th2, and Th17 CD4^+^ T cells as described in the material and methods section. (**A**) IFN-γ, (**B**) IL-4, and (**C**) IL-17A levels secreted from differentiated CD4^+^ T cells were measured using ELISA. The mRNA expression levels of (**D**) SOCS1, (**E**) SOCS3, and (**F**) IL-17A were analyzed using real-time PCR in differentiated Th17 murine CD4^+^ T cells. Data are mean ± S.E.M. values (*n* = 6). * *p* < 0.05, ** *p* < 0.01, *** *p* < 0.001.

Considering the suppressed systemic and regional IL-17A levels in the HWG-35D-treated group (Figure 3), we further investigated whether SK2 inhibition by HWG-35D directly affects Th differentiation of naïve CD4^+^ T cells. In this case, HWG-35D alters neither IFN-γ levels secreted from Th1-polarized cells (Figure 4A) nor IL-4 levels secreted from Th2-polarized cells (Figure 4B). In accordance with the previous studies with ABC294640 [21], IL-17A levels secreted from Th17-polarized CD4^+^ T cells were significantly decreased by HWG-35D (Figure 4C). These results indicate that SK2 inhibition by HWG-35D reduces Th17 differentiation of naïve CD4^+^ T cells. To elucidate the mechanism involved in this phenomenon, we examined SOCS1 and 3 expression levels, which are critical regulators of Th development [31,32]. Consistent with our previous report with ABC294640 [21], HWG-35D diminished SOCS1 mRNA levels significantly without altering SOCS3 transcription (Figure 4D and E). IL-17A mRNA levels were also reduced significantly by HWG-35D under Th17 polarization conditions (Figure 4F). These results suggest that blocking the generation of S1P by SK2 might abrogate Th17 development via reducing SOCS1 transcription.

### 2.6. The Protective Effects of Topical HWG-35D Application in Psoriasis-Like Skin Disease Were Investigated With the Dosage of 12.5 µG/40 µL Surfactant

**Figure 5:**
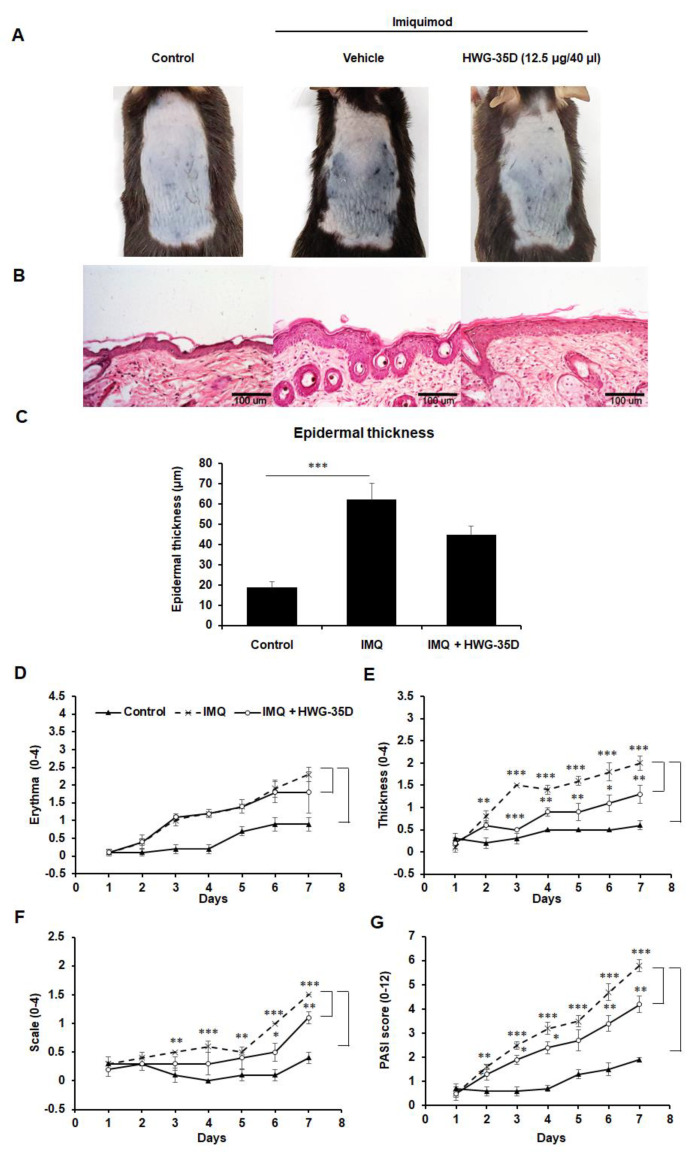
Topical application of HWG-35D with a dosage of 12.5 µg/40 µL surfactant only partially alleviates psoriasis-like skin disease. C57BL/6 mice were treated once daily with IMQ cream or the control vehicle cream on the shaved back for 6 consecutive days. HWG-35D treatment (12.5 µg/40 µL surfactant) began at 3 days before application of IMQ cream and sustained during IMQ treatment. (**A**) Phenotypical presentation of mouse back skin after 6 days of treatment. (**B**) Haematoxylin and eosin-stained skin tissue sections, and (**C**) quantification of epidermis thickness in the skin sections. The severity of (**D**) erythema, (**E**) thickening and (**F**) scaling were analyzed independently using the PASI score. (**G**) The comprehensive PASI score was represented. Data are mean ± S.E.M. (*n* = 5). * *p* < 0.05, ** *p* < 0.01, *** *p* < 0.001. The representative images are shown of three independent experiments (magnification ×20).

**Figure 6:**
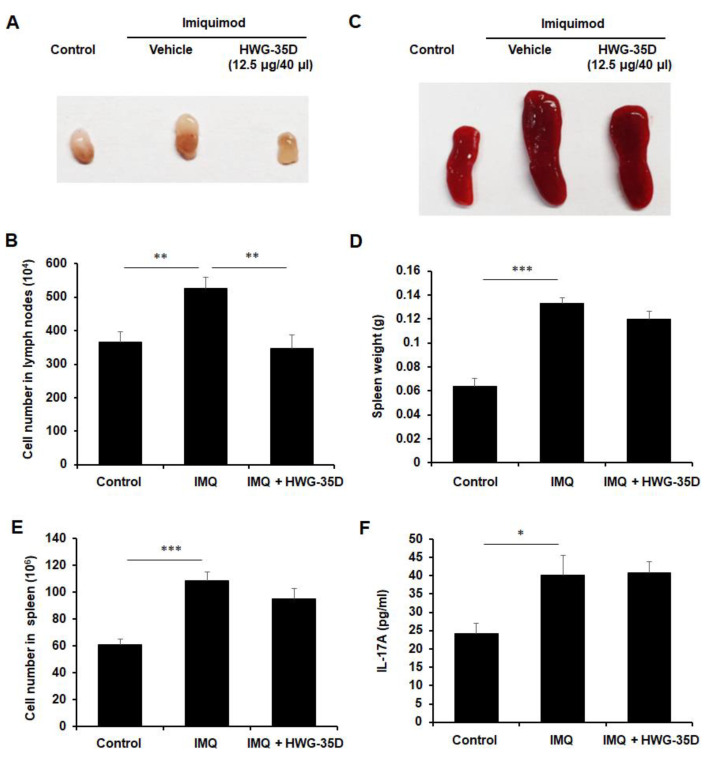
Topical application of HWG-35D with a dosage of 12.5 µg/40 µL surfactant only partially normalizes systemic immune response. (**A**) Gross morphologic examination of inguinal lymph nodes, and (**B**) their total cell numbers were counted in each group. (**C**) Gross morphologic features, (**D**) weights, and (**E**) total cell numbers of spleen were examined. (**F**) Serum IL-17A levels were examined using ELISA. Data are mean ± S.E.M. values (*n* = 5). * *p* < 0.05, ** *p* < 0.01, *** *p* < 0.001.

We also examined whether HWG-35D might mitigate psoriasis-like skin disease with the 12.5 µg/40 µL surfactant, which is the half dosage used in Figure 1. Although IMQ-induced psoriasis-like skin inflammation showed a tendency towards improvement (Figure 5A), histological reduction of epidermal thickness by HWG-35D at this reduced dosage was not statistically significant in H&E-stained skin sections (Figure 5B,C). The PASI score was applied to monitor daily skin changes of each group. Although erythema was not improved by HWG-35D (Figure 5D), thickness and scaling were significantly mitigated (Figure 5E,F), resulting in the effective reduction of the total PASI score in the HWG-35D-treated group (Figure 5G). To investigate whether HWG-35D suppresses the systemic immune response caused by IMQ, lymphoid organ size and cellularity was evaluated (Figure 6). IMQ treatment increased the sizes and mean cell numbers of the inguinal lymph nodes and this was normalized by HWG-35D using the 12.5 µg/40 µL surfactant dosage (Figure 6A,B). However, the corresponding changes in the spleen induced by IMQ were not significantly suppressed by HWG-35D at the 12.5 µg/40 µL surfactant dosage (Figure 6C–E). Finally, HWG-35D at the dosage of 12.5 µg/40 µL surfactant did not reduce serum IL-17A levels enhanced by IMQ (Figure 6F). These data indicate that topical application of HWG-35D at the 12.5 µg/40 µL surfactant dosage achieved only partial improvement of systemic and regional responses in psoriasis-like skin disease. 

## 3. Discussion

Psoriasis is mainly mediated by the effector cytokines secreted from Th17 cells, which activate the STAT3 signaling in keratinocytes, thereby inducing their hyper-proliferation and the expression of pro-inflammatory cytokines, growth factors, anti-microbial peptides and matrix metalloproteinases that cause chronic inflammation [6,33]. Recently, an important role for SK2 in Th17 polarization and psoriasis has been proposed [21,34]. However, these studies were conducted using ABC294640 and SKI-II [21,34], which have several other effects additional to SK2 inhibition [24,35,36]. Both ABC294640 and SKI-II have been reported to induce the ubiquitin-proteasomal degradation of SK1 and DES1 or lysosomal degradation of SK1 [24,35,36]. In addition, ABC294640 has been reported to be an estrogen receptor antagonist [15]. Therefore, the effects of ABC294640 and SKI-II on Th17 polarization might involve protein signaling networks regulated by proteins other than SK2. To exclude this possibility and confirm the effects of SK2 inhibition on Th17 polarization and psoriasis, we used a more selective and potent SK2-selective inhibitor, HWG-35D [17].

Previous studies mapping SK2 sequence differences onto the SK1/PF-543 crystal structure revealed subtle structural differences in the foot of the lipid-binding “J-channel” in SK2. HWG-35D (compound 55 in Adams et al. [17]) was synthesized as a potent SK2-selective inhibitor by replacement of the sulfonyl linker in PF-543 with a methyleneoxy linker and addition of a para-chloro group to the terminal phenyl ring [17]. The selectivity for inhibition of SK2 over SK1 is 100-fold for HWG-35D; IC_50_ for SK1 is 4130 nM and IC_50_ for SK2 is 41 nM [17]. In addition, HWG-35D is significantly more potent as an SK2 inhibitor compared with other SK2 inhibitors including ABC294640 (*K*_i_ 9800 nM) and SKI-II (*K*_i_ 6700 nM) [17]. Moreover, HWG-35D neither induces SK1 nor DES1 degradation. Therefore, HWG-35D is an excellent SK2 inhibitor without having an effect on SK1 or DES1. In addition, HWG-35D did not induce SK2 protein degradation in both human and murine cells, indicating that HWG-35D is a selective SK2 inhibitor, which does not affect its protein levels. The proteasomal degradation of human SK1 is mediated via Lys183 [37], and this residue is conserved as Lys182 in murine SK1. However, these residues are not conserved in human and murine SK2, and both residues are replaced with Arg313. According to the previous studies conducted by some of us, the topical application of ABC294640 with 25 µg/40 µL surfactant effectively alleviated psoriasis-like skin inflammation in vivo [21]. In the present study, topical administration of HWG-35D also improved psoriasis-like skin symptoms using the same dosage. These findings confirm that by using two structurally different compounds, namely HWG-35D and ABC294640, there is a protective effect of SK2 inhibition in psoriasis-like skin disease. In addition, efficacy was achieved with the half dosage (12.5 µg/40 µL surfactant) of HWG-35D compared with ABC294640. However, the establishment of which compound is more effective in vivo requires further study to establish the pharmacokinetics of each compound. Nevertheless, the alleviating effect of two chemically different SK2 inhibitors on the psoriasis-like skin disorder compared with ABC294640 alone provides stronger evidence for the pro-inflammatory role of SK2 in psoriasis. The present findings with HWG-35D also suggest that the effects of ABC294640 on psoriasis and Th17 differentiation are mainly mediated by SK2 inhibition rather than any possible off-target effects.

Psoriasis is one of the most common immune-mediated skin diseases characterized by hyper-proliferative keratinocytes and infiltration of immune cells [38]. Although the pathogenesis of psoriasis is not completely understood, the dysregulation of immune cells in the skin, particularly T cells, is regarded as a crucial mechanism for psoriasis progress [38]. Psoriasis was originally considered as a Th1-mediated skin disease [39]. Elevated levels of Th1 cytokines, such as IFN-γ, tumor necrosis factor-α (TNF-α) and IL-12 were observed in psoriatic lesions, whereas no such increase of Th2 cytokines (IL-4, IL-5, and IL-10) was observed [40,41]. However, keratinocyte proliferation is not directly induced by IFN-γ or TNF-α, and the pathogenesis of psoriasis is not fully explained by Th1 hyper-activation alone [41,42]. Recently, a key role for Th17 cells in psoriasis has been proposed [40]. Serum IL-17A, IL-22, and IL-6 concentrations were significantly higher in psoriasis patients compared with the control group [43]. Two monoclonal antibodies targeting IL-17A (secukinumab, ixekizumab) and one against the IL-17 receptor (brodalumab) are approved for the treatment of “moderate-to-severe” plaque psoriasis, and these treatments have shown high quality early responses and maintenance of clinical improvement in psoriasis patients [44]. In the present study, HWG-35D reduced Th17 differentiation from naïve CD4^+^ T cells. However, the compound did not affect Th1 and Th2 differentiation. Thus, HWG-35D might be effective for psoriasis, which is induced by the Th17 response. According to the previous report [34], SKI-II only reduces IL-17A production under Th17 polarizing conditions and does not affect IFN-γ or IL-4 production under Th1 or Th2 polarizing conditions, respectively. Therefore, the inhibitory effect of HWG-35D on Th17 differentiation is consistent with previous findings using ABC294640 [21] or SKI-II [34]. Together, these findings confirm an important role of SK2 in Th17 polarization. In the present study, SK2 inhibition using HWG-35D reduced SOCS1 levels during Th17 polarization. SOCS1 is critical in regulating Th17 differentiation by maintaining STAT3 and Smad transcriptional activities [32]. Indeed, Th17 differentiation is markedly reduced in SOCS1 deficient naïve CD4^+^ T cells or in SOCS3 overexpressed T cells [31]. Therefore, the data reported herein are consistent with the previous findings showing that ABC294640 treatment in naïve CD4^+^ T cells incubated in Th17 differentiating medium suppressed SOCS1 expression [21]. The exact mechanisms by which SK2 regulates Th17-dependent pathology and which have been described previously by some of us [12] requires further investigation. ABC294640 also binds estrogen receptors, acting as a partial antagonist similar to tamoxifen [15]. Although the effect of estrogen on Th17 polarization is controversial [45,46], the deficiency of estrogen receptor α can decrease Th17 cell differentiation by reducing IL-23 receptor expression on Th17 cells [47]. In the present study, IL-23 receptor mRNA levels were not significantly reduced upon HWG-35D treatment during Th17 polarization (data not shown). In addition, male mice were used in the present study and therefore the estrogen effect on Th17 differentiation would be negligible.

Although erythema was not improved with the half dosage (12.5 µg/40 µL surfactant) of HWG-35D, thickness and scaling were significantly mitigated. Psoriasis can be clinically classified into several types, including the most common psoriasis vulgaris, erythrodermic psoriasis, and pustular psoriasis with pus-filled, yellowish, and small blisters [48]. In erythrodermic psoriasis, erythema is involved at least 75% of the body surface area and the disease is associated with a predominantly Th2 phenotype [49]. Therefore, the relative lack of an effect on erythema with the half dosage of HWG-35D may be derived from the different mechanisms of each skin symptom.

High levels of systemic and local S1P have been reported in psoriasis patients [19,20], thereby highlighting S1P as a potential target for therapeutic intervention. HWG-35D is a highly selective SK2 inhibitor [17] and its ability to block ex vivo Th17-development, as shown here, and at concentrations as low as 100 nM (data not shown), suggest that the development and optimization of SK2 inhibitors with drug-like properties for treatment of psoriasis is warranted. The concentration of HWG-35D, which reduces Th17 differentiation in vitro is approximately 250 times lower compared with ABC294640 [22], and this therefore provides proof that it is possible to improve the potency at which SK2 inhibitors block Th17 polarization, which is necessary to translate SK2 inhibitors into effective medicines for the treatment of psoriasis. Moreover, the lack of effects on DES1 and SK1 suggests that side-effects related to these aspects of sphingolipid signaling can be limited for this class of inhibitor, as exemplified by HWG-35D.

## 4. Materials and Methods 

### 4.1. Materials

All materials were from Sigma-Aldrich (Sigma-Aldrich, St Louis, MO, USA) unless otherwise stated. IMQ cream (5%, Aldara) was purchased from Dong-Ah Pharmaceutical (Seoul, South Korea). HWG-35D was synthesized as described previously [17]. ABC294640 was from Cayman Chemical (Cayman Chemical, Ann Arbor, Michigan, USA). Anti-DES1, anti-SK1, anti-SK2 and anti-Ki67 were purchased from Abcam (Abcam, Cambridge, MA, USA). Anti-mouse-horseradish peroxidase and anti-rabbit-horseradish peroxidase antibodies were from Jackson Laboratory (Jackson Laboratory, Bar Harbor, ME, USA).

### 4.2. Cell Culture

MCF-7 (a human breast cancer cell line) cells were cultured in alpha-Modified Eagle’s Medium (α-MEM, Gibco; Thermo Fisher Scientific Inc., Waltham, MA, USA) supplemented with 10% fetal bovine serum (HyClone) and 1% penicillin/streptomycin (HyClone). NIH3T3 (a mouse embryonic fibroblast cell line) cells were cultured in Dulbecco’s Modified Eagle’s medium (Hyclone) supplemented with 10% bovine calf serum (Gibco) and 1% penicillin/streptomycin. HWG-35D (100–500 nM) or ABC294640 (25 μM) was added for 24 h to examine the degradation of SK1 and DES1. Cells were purchased from American Type Culture Collection (American Type Culture Collection, Manassas, VA, USA).

### 4.3. Animal Experiments

Eight-week-old male C57BL/6 mice were purchased from OrientBio (OrientBio, Seongnam, South Korea) and housed under specific-pathogen-free conditions with food and water ad libitum. To induce psoriasis-like skin lesions, 83 mg [50] of IMQ cream was topically treated on the shaved back region of mice once daily for six consecutive days as described previously [26]. The control group was treated similarly with a vehicle cream (Vaseline Lanette cream, Fagron, Rotterdam, Netherlands). To inhibit SK2 activity using HWG-35D, topical application of HWG-35D was started at 3 days before treatment with IMQ cream, and was maintained once daily with a dosage of 12.5 µg/40 µL surfactant (100% ethanol, propylene glycol, and H_2_O (EPH) at 2:1:1 *(v/v/v*)) or 25 µg/40 µL surfactant (Figure 2A). The dosage of 25 µg/40 µL surfactant was used to inhibit SK2 using ABC294640 in the previous study [21]. Control mice received the vehicle surfactant, EPH for three consecutive days before inducing psoriasis-like skin lesions with IMQ. All experimental procedures were approved in 20 June 2020 by the Animal Care and Use Committee of the Ewha Womans University School of Medicine (EUM-20-042).

### 4.4. Scoring the Severity of Psoriasis-Like Skin Lesion

As previously described [50], clinical Psoriasis Area and Severity Index (PASI) scores were used to evaluate the severity of inflammation of the back skin. Erythema, scaling, and thickening were measured with a scale from 0 to 4 (0, none; 1, slight; 2, moderate; 3, marked; 4, very marked), and the sum of each score represented the severity of skin lesion.

### 4.5. Histological Evaluation and Immunohistochemistry

For histological evaluation, mouse back skin samples embedded in paraffin after fixing with 4% (*w/v*) paraformaldehyde were sectioned at a 5-µm thickness. Haematoxylin and eosin (H&E) staining was performed according to standard methods. Epidermal thickness was measured using ImageJ software (NIH).

To perform immunohistochemistry, the fixed skin slides were sequentially stained with anti-Ki67 antibody (Abcam, Cambridge, UK) and horseradish peroxidase-conjugated secondary antibody after blocking endogenous peroxidase activity as described previously [21]. 3,3΄-Diaminobenzidine staining was conducted with the Vectastain elite ABC kit (Vector Laboratories, Burlingame, CA, USA) and the slides were counterstained with haematoxylin.

### 4.6. Enzyme-Linked Immunosorbent Assay

Enzyme-linked immunosorbent assays (ELISAs) were performed to determine IFN-γ, IL-4 and IL-17A levels in cell culture media of naïve murine CD4+ T cells using commercial kits from Biolegend (San Diego, CA, USA). Similarly, serum IL-17A levels, obtained at day 6 of IMQ treatment, were measured using the LEGEND MAX™ mouse IL-17A ELISA kit (Biolegend).

### 4.7. Real-Time Polymerase Chain Reaction

Extraction of mRNA from mouse back skin specimen or cell lysates was performed using RNeasy Mini kit (Qiagen, Valencia, CA, USA), and complementary DNA was synthesized from the mRNA using ReverTraAce qPCR RT master mix with gDNA remover (Toyobo, Osaka, Japan). Then, real-time polymerase chain reaction was performed with complementary DNA using an ABI PRISM 7500 sequence detection system (Applied Biosystems, Warrington, UK). According to the previous study [51], relative gene expression was calculated as 2^−ΔΔCt^ with normalization with the glyceraldehyde-3-phosphate dehydrogenase reference gene. The primer list is described in Table 1.

### 4.8. Western Blotting

Cells were lysed in radioimmunoprecipitation assay buffer (Tris–Cl (50 mM, pH 7.5), NaCl (150 mM), 1% Nonidet P-40, 0.5% sodium deoxycholate, 0.1% SDS and protease inhibitors). Protein samples (20 µg) from cell lysates were separated by 8% SDS-PAGE and then transferred to polyvinylidene difluoride membranes (Bio-Rad Laboratories). After blocking the membranes with 5% bovine serum albumin (Sigma-Aldrich, St Louis, MO) in phosphate-buffered saline with 0.1% Tween-20, membranes were incubated overnight (4 °C) in primary antibodies. Then, membranes were incubated with a relevant secondary antibody. Bands were detected using the ImageQuant™ Las 4000 mini biomolecular imager (GE Healthcare Life Sciences, Pittsburgh, PA, USA).

### 4.9. In Vitro Induction of T Cell Differentiation

Naïve CD4^+^ T cells were isolated from mouse spleen using Naive CD4^+^ T Cell Isolation Kit from Miltenyi Biotec (Gladbach, Germany). As reported previously [21], purified naïve CD4^+^ T cells were primed with plate bound anti-CD3 (1 µg/mL) (BD Biosciences PharMingen, San Diego, CA, USA) and soluble anti-CD28 (2 µg/mL) (BD Biosciences PharMingen) antibodies. For Th1 differentiation, the cells were cultured in the presence of soluble anti-CD28 (1 μg/mL), rmIL-2 (20 ng/mL; Peprotech, London, UK), rmIL-12 (10 ng/mL; R&D Systems, Minneapolis, MN, USA), and anti-IL-4 neutralizing antibody (10 μg/mL, Biolegend) for 5 days. For Th2 differentiation, the naïve CD4^+^ T cells were incubated in the media including soluble anti-CD28 (1 μg/mL), rmIL-2 (20 ng/mL), rmIL-4 (10 ng/mL; R&D Systems), and anti-IFN-γ neutralizing antibody (10 μg/mL, Biolegend) for 5 days. For Th17 differentiation, the cells were maintained in the media containing soluble anti-CD28 (1 μg/mL), rmIL-2 (20 ng/mL), rmTGF-β (5 ng/mL; R&D Systems), rmIL-6 (25 ng/mL; Peprotech), and anti-IL-4 and anti-IFN-γ neutralizing antibodies (10 μg/mL) for 5 days. The differentiated cells were transferred to a new plate to incubate for 2 days after extensive washing. Then, the cells were re-stimulated for another 40 h with Dynabeads Mouse T-Activator CD3/CD28 (Invitrogen, Carlsbad, CA) and supernatants were used to measure cytokine levels with ELISA. For SK2 inhibition, HWG-35D was used at 300 nM final concentration.

### 4.10. Statistical Analyses

Data are expressed as mean ± standard error of the mean (S.E.M.). Statistical significance was calculated using GraphPad PRISM 6 statistical software (GraphPad Software) to apply Student’s t test, one-way analysis of variance (ANOVA), or two-way ANOVA. *p* value < 0.05 was considered statistically significant.

## 5. Conclusions

In summary, the present study provides evidence for the role of SK2 in Th17 differentiation and suggests that highly potent and selective inhibitors of SK2 might be usefully translated to the clinic to treat psoriasis and other Th17-mediated diseases [46].

## Figures and Tables

**Table 1:** Primers used for real-time PCR.

Gene	Primer Sequence (5′-3′)	Reference
IL-17A	F: ATCAGGACGCGCAAACATGA	[52]
	R: TTGGACACGCTGAGCTTTGA	
IL-17F	F: GTCGCCATTCAGCAAGAAAT	[21]
	R: GGTGCAGCCAACTTTTAGGA	
K6	F: CTGGTAGTGGCTTTGGCTTC	[21]
	R: AGGCTCTGGTTGATGGTGAC	
K16	F: GGTGGCCTCTAACAGTGATCT	[53]
	R: TGCATACAGTATCTGCCTTTGG	
Gapdh	F: CACTCTTCCACCTTCGATGC	[54]
	R: CCCTGTTGCTGTAGCCGTAT	
SOCS1	F: CCTCCTCGTCCTCGTCTTC	[55]
	R: AAGGTGCGGAAGTGAGTGTC	
SOCS3	F: AGCTCCAAAAGCGAGTACCA	[56]
	R: AGCTGTCGCGGATAAGAAAG	

## Abbreviations

DES1	Dihydroceramide desaturase 1
ELISA	Enzyme-linked immunosorbent assay
EPH	Ethanol, propylene glycol and H_2_O
H & E	Hematoxylin and eosin
IFN	Interferon
IL	Interleukin
IMQ	Imiquimod
PASI	Psoriasis Area and Severity Index
SK	Sphingosine kinase
S1P	Sphingosine-1-phosphate
SOCS	Suppressor of cytokine signaling
STAT3	Signal transducer and activator of transcription
Th	T-helper
TNF-α	Tumor necrosis factor-α
